# Dr. Theophilus Thompson on the Application of the Microscope to the Diagnosis of Pulmonary Consumption

**Published:** 1857-11

**Authors:** 


*Dr. Theophilus Thompson on the Application of the Microscope to the Diagnosis of Pulmonary Consumption.*

The author observed that the assiduous and discriminating use of the microscope having yielded valuable aid in illustrating various pathological questions, and giving precision to some grounds of diagnosis, it seemed reasonable to anticipate information from the application of this instrument to the examination of expectoration; and he thought it practicable to show that the disappointment which some observers have experienced in their endeavors to avail themselves of this method of investigation did not depend on inadequacy of the means, but might be overcome by care and perseverance. Many years since, Mr. Quekett detected elastic pulmonary tissue, in the sputum of patients not previously considered consumptive, and it was for a time supposed that a peculiar granular appearance of the expectoration might be regarded as characteristic of tubercular disease, even in the absence of any trace of elastic tissue. Finding, after a time, that this appearance could not be relied on as an indication of incipient phthisis, and embarrassed by the multiplicity of objects often present in the expectoration, Dr. Thompson for a time discontinued the investigation, any sanguine expectations which he had entertained being further discountenanced by the testimony of Rainey, Addison and Bennett;* but in an interview with Dr. Andrew Clark (to whose sagacious observation and faithful descriptions† the profession is greatly indebted), he had the gratification of learning that the subject had engaged his attention since the year 1846, and with such success as to enable him to show, in his lectures at Haslar, the real microscopical indications of tubercular

* Dr. Hughes Bennett has lately added his testimony to the value of the microscope in suspected phthisis. Vide Edinburgh Monthly Journal, Jan., 1856, p. 585.

† Vide Transactions of the Pathological Society, vol. 6, p. 74; and Lettsomian Lectures, by Theophilus Thompson, M.D., F.R.S.

sputum. With his friend's liberal and courteous assistance, Dr. Thompson soon became convinced that changes in the pulmonary vesicles, preceding the stage of destruction which occasions the elimination of pulmonary tissue, are manifested in the expectoration, and that information may thus be obtained, not only supplying valuable aid in diagnosis, but also furnishing instructive information regarding the morbid process concerned. Dr. Thompson showed, by a diagram enlarged from a drawing by Shrœder Van der Kolk, that when tubercular deposit is present in the pulmonary vesicles, there may be seen, contrasting with the usual epithelial cells, some which are dark, swollen, spherical; some more advanced, larger and misshaped; others shrivelled or burst, and extruding nuclei, which nuclei, when enlarged, correspond with the "tubercle corpuscles" of Lebert. The author proceeded to show that the sputum of consumptive patients contains materials corresponding in appearance with the elements present in the air-vessels, and that before an amount of disease involving the elimination of elastic areolæ occurs, corpuscles of various sizes, jagged outline, setting free nuclei, and affording evidence of rapid disintegration may be detected. The general moleculo-granular appearance (to which his attention had been originally directed, and which he much regretted having erroneously figured in his "Clinical Lectures") was not conclusive; the sputum, which is really characteristic, containing isolated masses of moleculo-granular material, and having interspersed corpuscles of various forms, overgrown or jagged, and setting free nuclei; the various proportions of pus, or fat, or blood, giving collateral indications of the amount of surrounding deterioration in the lungs; while amongst evidences of rapid progress might be specified the appearance of large and numerous areolar meshes, still retaining their adhesion and elasticity. In chronic cases, portions of this tissue appear, inelastic, teased out, and broken down, in consequence of long imprisonment, whilst a diminished proportion of fat, and the appearance of cholestrine plates, and still more of earthy particles, were often indicative of a mode of restoration. The author proceded to prove, by a brief narration of cases:

First, that with the aid of the microscope positive conclusions, not attainable by auscultation, could sometimes be formed regarding the existence of pulmonary disease.

Case 1.—Mr. ——, aged sixty-three, after an attack of pleurisy in the left side, during the spring of 1855, did not regain strength. Dull percussion and prolonged expiratory murmur over a small portion of the right apex were the only important auscultatory signs; but the expectoration, under the microscope, was found to contain blood corpuscles, moleculo-granular matter, and lung-tissue broken down and unbent. More positive symptoms of decided phthisis, as reported by his medical attendant in the country (Dr. Sylvester of Trowbridge), soon appeared, and in a few months he died.

CASE 2.—Mrs. E——, a lady, aged thirty-nine, whom Dr. Thompson attended with Mr. Marshall of Bedford-square, during the early months of 1855, suffered from obstinate sickness, and the progressive emaciation inducing an examination of the chest, some dulness on precussion with increased vocal thrill was observed near the sternal end of the second intercostal space on the right side. A little expectoration was obtained, and was found to contain shrivelled cells, lung-tissue, and isolated masses of granules. Some improvement of the general health occurred under soothing hygienic and tonic treatment, and the administration of cocoblein. But early in the year 1856 the expectoration became copious and flocculent; dulness on percussion was more extensively obvious; near the inferior angle of the scapula click was audible, shortly followed by cavernous breathing. In March she died. An interesting contrast to this story was afforded by

CASE 3.—A lady, aged thirty-eight, who, in the autumn of 1852, had almost precisely the same auscultatory symptoms as were observable in Mrs. E——; but the occasional, slight, cloudy expectoration, from time to time examined, exhibited ciliary cells, some with long tails, probably tracheal, some in masses, as though from the follicles; but there were no tubercular elements. In harmony with the encouraging testimony thus afforded by the microscope, the general symptoms continue favorable, and have hitherto, during a period of five years, negatived the gloomy prognostications which an accomplished auscultator had perseveringly maintained.

Secondly: the author adduced the advantage of microscopical observation in confirming doubtful signs.

CASE 4.—E. T——, aged fifty-one, in the winter of 1854 was attacked with cough, hurried breathing, and some symptoms of hectic. The left lung had been extensively consolidated in consequence of pleuro-pneumonia ten years previously. Over a small space near the lower angle of the left scapula a sound could be heard, of which it was difficult to determine whether the correct designation were subcrepitation on click. Dr. Andrew Clark, who also obligingly examined the expectoration, reported that it contained shrivelled cells, large cells with shrivelled nuclei, and some earthy matter, and, without receiving any history of the case, offerred the diagnosis of "Slight tubercular deposit, tending to restoration," a diagnosis which was confirmed by the result.

Thirdly: Dr. Thompson described some favorable indications afforded by the microscope concurrently with amelioration in the general condition.

CASE 5.—Mr. ——, aged twenty-two (introduced by Mr. Pinching, of Gravesend), five feet nine inches in height, in the summer of 1854 had dull percussion and a murmur over the left pulmonary artery, but no crackle or click; the expectoration, however, exhibited lung tissue, tubercle corpuscles, and blood-discs. He took cod-liver oil freely, at one period to the extent of a pint and a half in a week; and had ioduretted neat's-foot oil (a grain to the ounce) rubbed into the chest. After a time the expectoration became chiefly bronchial, disposed to fibrillate, and free from lung tissue. The weight of this patient increased fron ten stone one pound to eleven stone nine pounds. He spent last winter in Madeira.

Fourthly: The author noticed the important evidence sometimes derivable from the sputum, indicative of rapidity in the progress of disease.

CASE 6.—A lady in the country, aged forty-three, who had been for two years the subject of phthisis, but whose friends did not fully realize the danger, had a decided aggravation of cough and weakness. Some expectoration, sent to town for examination, contained blood, copious pus corpuscles, and numerous large meshes of pulmonary tissue, perfectly retaining their form and elasticity. A very unfavorable prognosis was consequently given, which was verified by the death of the patient a few days afterwards.

The author in conclusion, ventured to express the opinion that his statements, although brief, were sufficient to support his proposition, that the microscopical inspection of expectoration might often afford, at a very early period of consumption, definite information, not otherwise attainable, regarding the nature of the malady; that in later stages of disease it might assist us to estimate the rapidity and progress, and at all times might furnish valuable aid in forming a correct prognosis regarding the course of the complaint. He trusted these few suggestions would stimulate to the investigation some of his professional brethren more accomplished in the use of the microscope, or more fortunate in the enjoyment of leisure.—*Lon. Lan.*

